# Malignant Transformation of a Sebaceous Cyst Into Squamous Cell Carcinoma in an Elderly Male Patient

**DOI:** 10.7759/cureus.91449

**Published:** 2025-09-02

**Authors:** Harsh V Baranwal, Himanshu Panwar, Ram Niwas Meena, Rahul Khanna

**Affiliations:** 1 General Surgery, Institute of Medical Sciences, Banaras Hindu University, Varanasi, IND

**Keywords:** chest wall tumor, malignant transformation, sebaceous cyst, squamous cell carcinoma, surgical case reports

## Abstract

Malignant transformation of sebaceous cysts into squamous cell carcinoma (SCC) is exceptionally rare. We report the case of a 65-year-old man with a long-standing anterior chest wall sebaceous cyst that ulcerated and rapidly enlarged over three months. Examination revealed a fungating lesion without chest wall invasion. The patient underwent wide local excision with adequate margins and primary closure, recovering uneventfully. Histopathology confirmed moderately differentiated SCC. This case underscores the importance of early biopsy and surgical management when chronic cystic lesions develop ulceration, rapid growth, or atypical changes.

## Introduction

Sebaceous (epidermal inclusion) cysts are common benign cutaneous lesions, typically presenting as slow-growing nodules filled with keratin or sebaceous material. Malignant transformation of epidermal (sebaceous) cysts into squamous cell carcinoma (SCC) is exceedingly rare, with reported incidence ranging from approximately 0.011 % to 0.045 % in the literature [1,2]. Since the first documented cases, fewer than 50 well-characterized instances of SCC arising from epidermal cysts have been reported, most commonly on the head, neck, trunk, and perineum [3-5]. Risk factors implicated include chronic inflammation, repeated infection, mechanical irritation, ultraviolet exposure, and potentially HPV infection [4,5]. Clinically, red flag signs such as sudden enlargement, ulceration, bleeding, foul discharge, or pain should prompt early biopsy to exclude malignancy [5,6]. We present this case to highlight the importance of maintaining a high index of suspicion for malignant change in long-standing sebaceous cysts, particularly when new symptoms such as ulceration, rapid enlargement, or foul discharge occur. Histopathologically, these tumors often derive from cyst wall epithelium and display atypical squamous proliferation, pleomorphism, keratin pearl formation, and nuclear hyperchromasia [7]. This report also addresses the limited documentation of chest wall lesions of this nature in the literature. 

## Case presentation

A 65-year-old man presented to the surgical outpatient department with a large, ulcerated, exophytic lesion over the anterior chest wall, approximately at the level of the fourth intercostal space (T4 dermatome). The patient had a history of a stable, painless swelling at the same site for over eight years, which had periodically discharged a thick, cheesy sebaceous material. Four months prior to presentation, the lesion ulcerated spontaneously, followed by rapid growth in size over the last three months, becoming painful and foul-smelling.

**Figure 1:**
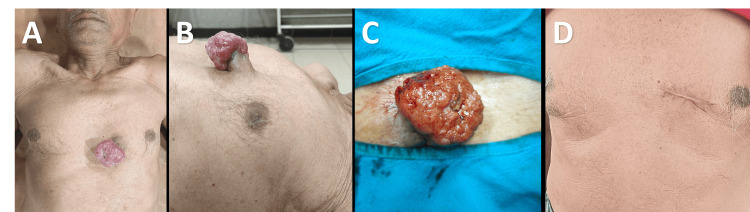
A. Preoperative clinical image showing an ulcerated, fungating lesion over the anterior chest wall at T4 dermatome. B. Side view of the lesion demonstrating a broad base and a necrotic surface. C. Close up of the lesion prior to wide local excision. D. Follow-up at six weeks.

On examination, there was a 6 × 5 cm fungating, cauliflower-like mass arising from the skin with a granulated surface showing areas of necrosis and serosanguinous discharge (Figure 1). The lesion was not fixed to deeper tissues, and no palpable axillary or cervical lymphadenopathy was noted.

Laboratory parameters were within normal limits. The patient had no significant comorbidities such as diabetes, immunosuppression, or chronic skin conditions. Systemic examination was unremarkable. There was no evidence of other skin lesions or suspicious lymphadenopathy on general examination. CECT thorax showed no evidence of nodal, rib, pleural, or lung involvement.

**Figure 2:**
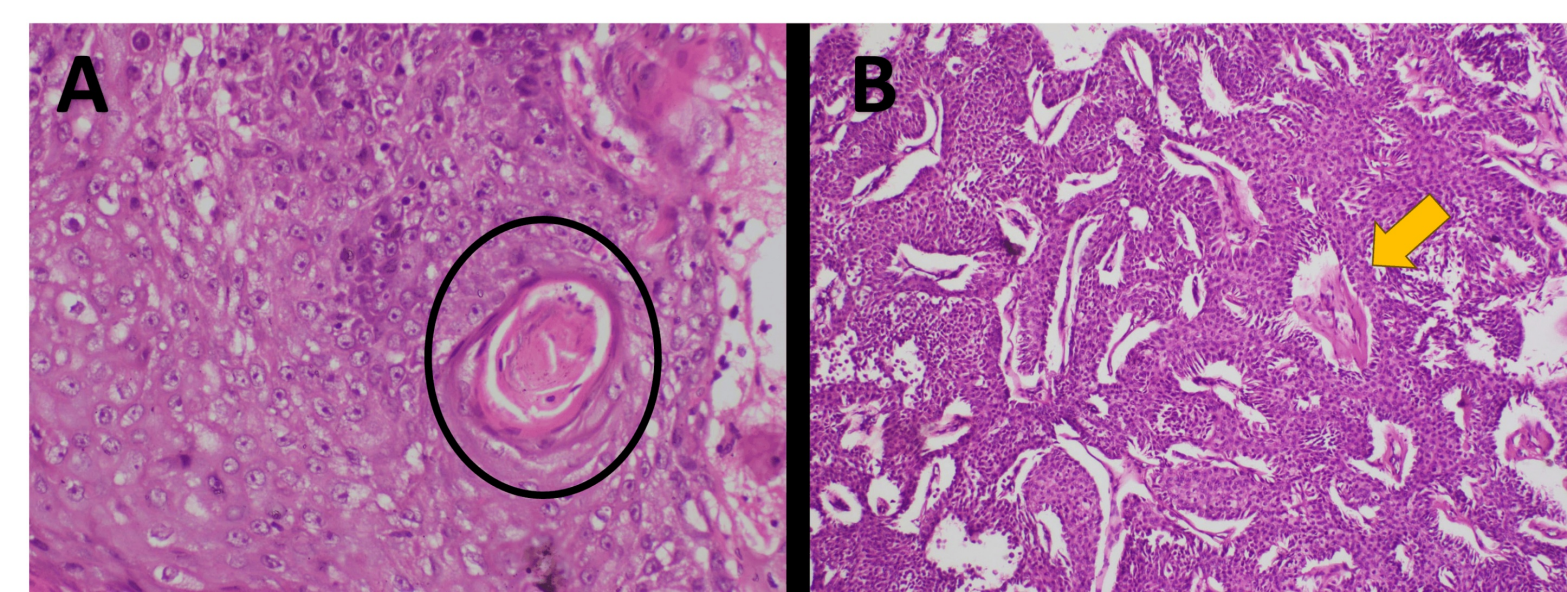
A. Histopathology (H&E, ×200) showing moderately differentiated SCC with keratin pearl (black circle). B. Histopathology (H&E, ×20) showing polygonal spindle-shaped cells with hyperchromatic pleomorphic nuclei, coarse chromatin, and prominent nucleoli with a moderate amount of cytoplasm. Areas of hemorrhage and keratinization (yellow arrow) are seen. H&E: Hematoxylin and Eosin; SCC: Squamous Cell Carcinoma

The patient underwent wide local excision with 2 cm margins under general anesthesia, followed by primary closure. Histopathological examination confirmed a moderately differentiated keratinizing squamous cell carcinoma with neoplastic squamous nests infiltrating beyond the cyst wall, accompanied by keratin pearl formation; the polygonal cells exhibited hyperchromatic pleomorphic nuclei, coarse chromatin, prominent nucleoli, and moderate cytoplasm, with areas of hemorrhage and keratinization. All surgical margins were negative, and staining was performed with hematoxylin and eosin after formalin fixation and sectioning (Figure 2).

Follow-up

At six weeks and 4.5 months, wound healing was complete and no recurrence or nodal spread was detected. The patient is kept on regular follow-up every three months.

## Discussion

SCC arising from sebaceous cysts remains an exceptionally rare entity, with fewer than 50 well-documented cases reported in the literature, the majority involving the head, neck, or trunk. This case adds to the sparse documentation of anterior chest wall presentations (Table 1).

**Table 1:** Key features of previously reported SCC arising from sebaceous cysts with the present case, including patient demographics, site, duration, treatment, and outcomes. WLE: Wide Local Excision; SCC: Squamous Cell Carcinoma

Author, Year	Site	Duration of Cyst	Clinical Change	Treatment	Outcome
Chiu et al., 2007 [1]	Left thigh	40 years	Rapid enlargement	WLE	No recurrence at two-year follow-up
Park et al., 2018 [3]	Perineum	30 years	Rapid enlargement, ulceration	WLE	No recurrence at three-year follow-up
Sumi et al., 2012 [5]	Perineum	8 years	Rapid enlargement	WLE	No recurrence at five-month follow-up
Present case	Chest wall	8 years	Ulceration, rapid growth, discharge	WLE	No recurrence at 4.5 months follow-up

In the present case, a 65-year-old male patient with an eight-year history of a sebaceous cyst localized to the T4 dermatome experienced ulceration four months prior, followed by rapid growth over the subsequent three months, mirroring classic warning signs associated with malignant transformation in a similar case by Sumi et al. [5].

Histopathologically, SCC arising from epidermal cysts demonstrates neoplastic squamous nests infiltrating beyond the cyst wall, often producing keratin pearls and cellular atypia [3]. Although metastatic spread remains uncommon (3-15%), aggressive behavior has been documented in select cases [5].

The histopathological hallmark of malignant transformation in cysts includes keratin pearl formation, nuclear pleomorphism, and invasive squamous nests emerging directly from the cyst wall without overlying epidermal involvement [8]. Although data are limited, surgical excision with negative margins (R0 resection) is considered curative in the majority of reported cases [9]. Recurrence has been noted, particularly when margins are close or positive-especially in lesions exceeding 3 cm, highlighting the importance of wide local excision [8,9]. Clinical warning signs prompting biopsy or excision include rapid growth, ulceration, fistula formation, and lesion size >5 cm [2,5].

Mohs micrographic surgery may be considered for lesions in cosmetically sensitive regions, and adjuvant radiotherapy is indicated for cases with positive margins or nodal involvement [1,5]. In our case, wide local excision with a margin of 2 cm was done. No radiotherapy was given as there was no nodal involvement or positive margin in histopathology.

Overall prognosis for SCC derived from epidermal cysts is favorable when diagnosed early and excised completely; however, vigilant long-term follow-up is advised due to occasional reports of recurrence and metastasis [2,5]. This highlights the importance of not dismissing long-standing cutaneous lesions as benign solely based on chronicity. Even clinically indolent cysts, especially those exposed to repeated irritation or located in high-friction areas like the trunk, can undergo neoplastic transformation. Increased awareness and clinical suspicion among surgeons and dermatologists can lead to earlier biopsy and intervention, improving outcomes.

## Conclusions

SCC arising from a sebaceous cyst is an uncommon but important diagnostic consideration when long-standing cystic lesions develop rapid growth, ulceration, or atypical features. This case demonstrates that prompt clinical suspicion, timely histopathological confirmation, and early surgical intervention with adequate margins can result in favorable outcomes and prevent local recurrence. Clinicians should maintain vigilance for malignant change in chronic skin lesions, particularly in elderly patients, to enable early detection and definitive management.
